# Experimental Infection with Sporulated Oocysts of *Eimeria maxima* (Apicomplexa: Eimeriidae) in Broiler

**DOI:** 10.1155/2014/283029

**Published:** 2014-08-26

**Authors:** Luciana da S. Brito, Elder N. Pereira, Augusta A. da Silva, Vinícius Bentivóglio Costa Silva, Fagner L. da C. Freitas

**Affiliations:** ^1^Anatomy Laboratory, Institute of Biotechnology and Health, Federal University of Amazonas, Road Coari/Mamia, n° 305, 69460-000 Coari, AM, Brazil; ^2^Laboratory of Parasitology, Federal University of South Border, Campus Royalty, Rua General Osório 413d, Italy Garden, 89802-210 Chapecó, SC, Brazil

## Abstract

Through this study we assessed the metabolic and pathological changes in broilers experimentally infected with oocysts of *Eimeria maxima*. To perform the experiment, we used 150 broiler strain cooB males, with ten days of age, were randomized according to weight and randomly assigned to two experimental groups: the control group was inoculated with 0.5 mL of distilled water; the infected group inoculated with 0.5 mL of solution containing 5 × 10^4^ sporulated oocysts of *Eimeria maxima*. The live performance was evaluated on day 0 (day of inoculation), 5°, 10°, 15°, 25°, and 35° dpi, being slaughtered by cervical dislocation, fifteen birds/group. Although the sum in meat production was higher in the control group, the weight of the heart and gizzard of the experimental animals showed no significant difference, while the liver had difference on day 5°, 15°, and 35° dpi. The pathologic evaluation showed congested mucosa and presence of large amounts of mucus at 6 dpi. Therefore, it is concluded that the dose of 5 × 10^4^ *E. maxima* inoculated in the experimental group was enough to cause harm to the animal organism.

## 1. Introduction

In recent decades there has been an increase in consumption of chicken meat in Brazil and in the world, and it has forced the development of technologies to be employed in industrial poultry production to improve food and earliness of chickens that are taken for slaughter [1].

The chicken meat consumption is increasing in Brazil. The average in 2011 was 47 kilograms, and in 2010 it was 44 kg per capita, winning the third place in the global ranking of per capita chicken meat consumption, just behind the United States and Saudi Arabia [2]. According to Avisite [3], preliminary data from USDA indicate a volume of just over four (4) million tons in chicken meat exporting, which represents an increase of 4% compared with the year 2009.

Even with the use of high technology, the intensive system of poultry production does not ensure that the poultry production environment is free of pathogens that harm the efficiency of utilization of nutrients as a result of the probable enteric disorders onset [4].

The main parasites found in poultry is caused by protozoa of the genus* Eimeria*, also known as coccidia, being responsible for serious economic losses, mainly due to diarrhea and deaths in young animals [1, 5].

The eimerioses present an endemic character in poultry farms, being descripted in seven* Eimeria* species that cause coccidiosis in poultry:* E. acervulina, E. praecox, E. maxima*,* E. mitis, E. necatrix, E. tenella, *and* E. brunetti* [1, 6, 7].

The agents that cause coccidiosis are intracellular parasites that are multiplied in the intestine, causing tissue destruction and harming digestion and absorption of food, resulting in clinical watery or bloody diarrhea [8]. For Allen and Fetterer [9], clinical signs of coccidiosis vary according to the species of coccidia present in the infection. The* Eimeria maxima* is moderately pathogenic and considered among the remaining species as the most immunogenic [10, 11], leading to decrease in weight gain, high morbidity, diarrhea, and, rarely, death [12, 13]. Producers interested in maintaining a good skin color, as well as creations of laying hens, should be concerned with subclinical infections because the effect of this kind is related to the absorption of xanthophyll carotenoid pigments for the small intestine [14].

Goodwin et al. [15], studying the different detection methods for* E. maxima*, concluded that the method of evaluation by lesion score individually does not confer great diagnostic value; the degree of histopathological injury is the best diagnostic method followed by microscopic oocyst count.

The study aims to examine pathological and metabolic disorders in animals experimentally infected with oocysts of* Eimeria maxima*.

## 2. Materials and Methods

The research was conducted at the School of Veterinary Medicine and Animal Science of the Federal University of Tocantins (UFT), located in the municipality of Araguaína, TO, while the experimental protocol and animal husbandry analyses were performed at the Histology Laboratory of the Federal University of Amazonas (UFAM), Manaus, AM, in which the creation and analysis of histological slides were performed, and at the clinical laboratory of the Regional Hospital of Coari, from Coari, AM, where the biochemical analyses took place.

To perform the study, 150 Coob male broilers with ten days of age were used. A suspension containing 1 × 10^4^ sporulated oocysts of* E. maxima* per mL of solution obtained from a pool of samples farm in Brazil was used. The birds were randomized according to weight and divided into two experimental groups: control group was inoculated with 0.5 mL of distilled water and the infected group was inoculated with 0.5 mL of solution containing 5 × 10^4^ oocysts of* Eimeria maxima*. For inoculation, all birds in the trial were included and manually inoculated with the aid of an automatic pipette by mouth.

Each group was housed in iron cages with dimensions of 1 m long × 2 m wide × 1.5 m high. The feeders and water dispensers used, respectively, containing clean water and balanced diet without anticoccidial were of the gutter type, being washed with water and neutral detergent, and flamed every 12 hours so that the risk of reinfection was avoided. The composition of feed used (based on corn and soybean meal) followed the rules and nutritional standards established by the NRC (1998), being supplied to the birds* “ad libitum.”*

During the experimental period, daily clinical examinations were conducted in animals and parasitological analysis was conducted in their feces; by means of isolation flotation technique, the sample was collected with the aid of spatula and stored in plastic containers and subsequently analyzed to determine the periods of prepatency (time between the time of inoculation of the host and the detection of the agent in tissue, secretions, or excreta) and patency (time interval between the beginning of the removal of oocysts by the birds and the termination of infection)* E. maxima*.

The growth performance was evaluated on day 0 (inoculation), 5°, 10°, 15°, 25°, and 35° dpi, being slaughtered by cervical dislocation, fifteen birds/group as resolution of the Committee of Ethics and Animal Welfare UFAM (Protocol No. 008/2013). At the collecting body weight, carcass weight and carcass yield were evaluated, with a scale of 0.1 g precision being used.

In order to determine the feed conversion, feed intake was evaluated, being daily weighed before being given to birds in common trade balance with maximum weight of 25 pounds and an accuracy of 0.5 g. The calculation was made for the difference in weight between the supplied food and its consumption. This value was derived by deducting the remnants of the total feed ration provided during the daily cleaning of feeders.

For the determination of carcass yield, the chickens were subjected to six hours of fasting and slaughtered by decapitation between the occipital bone and atlas using previously sterilized knife. The birds were bled for 2 minutes in a cone adapted to slaughtering chickens. Removal of the skin and feathers was manually done, and the birds were eviscerated by abdominal cut made with scissors. For calculating carcass yield, the weight of the eviscerated carcass was used (without viscera, feet, head, and abdominal fat). In relation to live weight, feed conversion ratio was estimated by food consumption (kg/bird/day) divided by weight gain (kg/bird/day).

Histopathologic evaluation was performed at the Laboratory of Histology, Department of Morphology, UFAM, where fragments of duodenum, jejunum, and ileum at 5, 10°, 15°, 25°, and 35 dpi were harvested and collected, opened longitudinally, washed in buffer phosphate (0.1 M, pH 7.4) and fixed in 10% formalin solution for 36 hours, then washed in 70% ethanol to remove the fixative and subsequently dehydrated in a series of increasing alcohol, and cleared in xylene and embedded in paraffin. After the semiserial microtome to a thickness of 7 *μ*m 7 histological sections were placed on each slide being stained with hematoxylin and eosin and observed at light microscopy [16].

To evaluate the hematocrit and metabolism of lipids, proteins, and carbohydrates, 5 mL of blood were collected after fasting for 6 hours intracardiac route, using syringes and needles being deposited in one ml sterile test tubes containing acid sodium ethylene diamine tetraacetic (EDTA-sodium), 1 mL in sterile test tubes containing ethylene-diaminetetraacetic acid sodium fluoride (EDTA-fluoride) and to 3 mL in sterile test tubes without anticoagulant to obtain whole blood, plasma and serum, respectively. Determination of plasma glucose was performed by the enzymatic method and the overall scores obtained by centrifugation in hematocrit capillaries were determined. Serum lipids were assessed in serum: lipoprotein triacylglycerols and total cholesterol.

The protein metabolism was assessed using total protein concentrations. All related biochemical parameters were determined by means of commercial kits (Labtest Systems Diagnostics Ltd— Belo Horizonte/MG).

The data were presented in tables, where average and the standard deviation were calculated (SD) because the data presented homogeneous and normal results at 5% level of significance by means of Bartlett's and Shapiro-Wilk tests, respectively. In average comparison, Student's *t*-test was applied [17, 18].

The software used in the analysis was the Epi-Info version 7 for Windows program developed and freely distributed by the CDC (http://wwwn.cdc.gov/epiinfo/7/). Significance testing was fixed at 5%.

## 3. Results and Discussion

The infected group showed a prepatency and patency of 5 and 12 days, respectively, with two peaks of oocysts shedding. The first peak occurred at 5° dpi and remained constant until the 7th dpi, reaching maximum elimination of 52,000 oopg. After 7 dpi, there was a reduction in the number of oocysts eliminated. The second peak occurred on the 12th dpi, but the amount of oocysts released in the feces was lower (28,000 oopg) at the first peak. Importantly, the period of the greatest elimination of oocysts in the feces coincided with the days when the animals showed more severe clinical cases of diarrhea. After 6 dpi infected animals gradually returned feed intake until they reach the same proportion of animals in the control group. The end of the patent period was confirmed by parasitological stool examination and histopathological analysis of the small intestine. The control group remained negative for the presence of oocysts throughout the experimental period.

**Figure 1:**
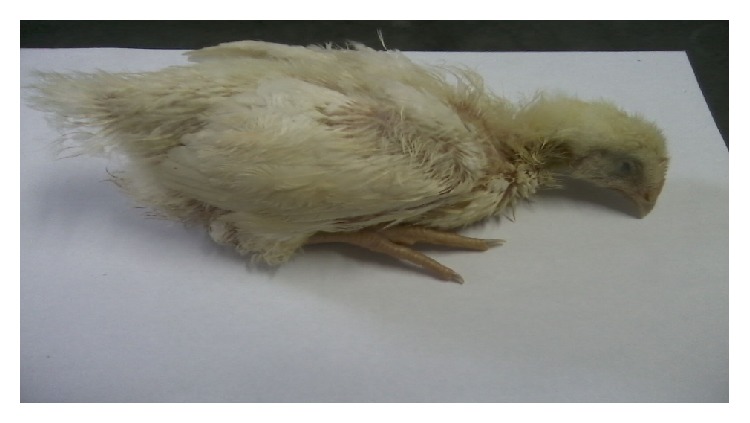
Infected bird, presenting physical weakness in the 6th dpi.

**Figure 2:**
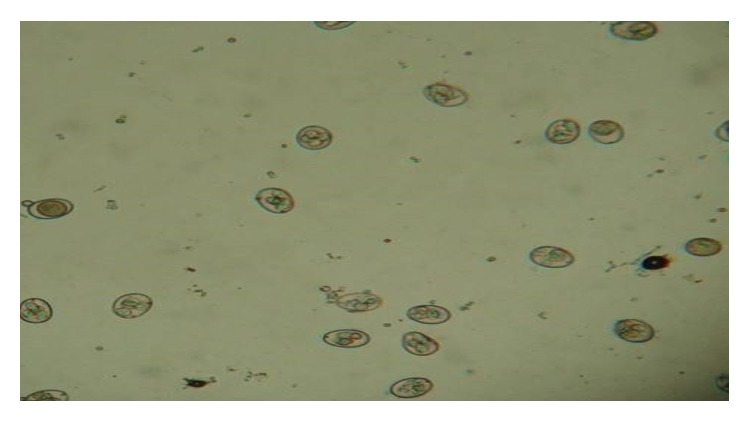
Isolated oocysts in the infected birds' feces, during the period of patency and sporulated “in vitro” to confirm its species.

The first clinical signs were seen in 5 dpi and the birds showed apathy followed by fetid diarrhea, mucoid, ruffled feathers, and less weight gain compared to the birds of the control group (Figure 1). The infected group showed a prepatency and patency of 5 and 12 days, respectively. The end of the patent period was confirmed by parasitological stool examination and histopathological analysis of the small intestine (Figure 2) and the control animals remained negative for the presence of oocysts throughout the experimental period. These characteristics are similar to findings in other studies with results of five- and seven-day period prescribed prepatent and patent [19–21].

**Figure 3:**
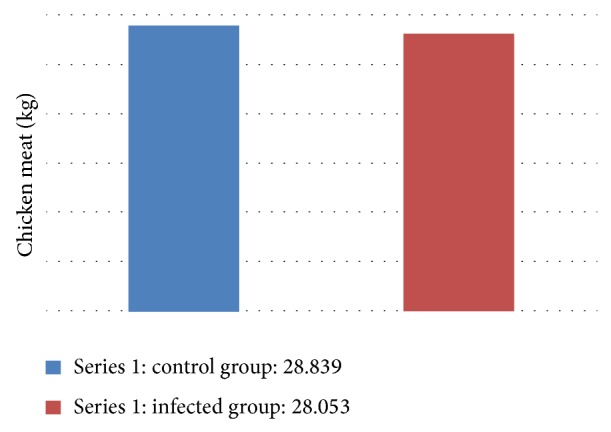
Meat Production Chart from the 5th to the 35th dpi, in terms of grams.

The prepatent period observed in this study differs from the results found by Zulpo [13], who studied the pathogenicity of strains of* E. maxima*, using a dose of 2 × 10^4^ and who found a prepatent period of 168 hours (7 dpi). Idris et al. [12] also noted a prepatent period of 168 hours in chickens inoculated with 2.5 × 10^4^ oocysts* E. maxima*. The lowest prepatency may be related to high excystation of sporozoites of* E. maxima*, according to Shiotani et al. [22]. At the end of the experiment, the control group produced 28.839 kg of meat, while the infected group produced 28.053 kg of meat (Figure 3). Silva et al. [23], evaluating the performance of broilers reared in environments with and without oocysts, observed a lower body weight and a lower feed at 14 days of life conversion. Already Persia et al. [24] reported that the coccidiosis infection can lead to a drop in performance due to decreased dietary metabolisable energy, and digestibility of amino acids.

The results of the average live weight, carcass weight, liver, heart and gizzard, and control groups infected in 10°, 15°, and 25° dpi are shown in Table 1.

Zulpo [13] did not obtain significant differences in weight gain, but always lower average for the infected group. Mori's work [25] corroborates to the previous one, mentioning that when using a single dose of 4 × 10² of live attenuated oocysts* E. maxima, E. acervulina, E. mitis, *and* E. tenella*, the infected group showed a decrease in all parameters compared to the control group. The weight of the heart and gizzard of the experimental animals showed no significant difference. On the other hand, the liver had difference on 5°, 15°, and 35° dpi. As for the data of hematocrit, the groups showed no significant difference (*P* > 0.05) from baseline to the end of the experiment, 5°–35° dpi; see Table 2.

Under the study conditions, significant difference (*P* < 0.05) was found in glucose levels in all collection days, favorable to the control group. Normal values for serum glucose may be linked to hepatic glycogenolysis performed by the bird, where glucose is maintained on its basal levels through reduced hepatic glycogen by glucagon release from the pancreas. According to Chapmanet al. [26], the ability of the bird has to maintain normoglycemia in situations where liver glycogen reserves are depleted, which is due to gluconeogenesis or a reduced utilization of glucose by it. Similar results were obtained by Chapman et al. [26] by inoculating higher dosage of oocysts of* Eimeria maxima* than that used in this experiment, which observed that glucose concentrations remained similar between the infected and the control group. Divergent results were found by Ruff et al. [27], studying glucose uptake in birds infected with* E. maxima*, where he observed that it causes changes in the absorption of glucose in the acute phase of the disease. However, at 21 days postinfection changes in plasma glucose concentrations were not observed.

Protein levels remained significant (*P* < 0.05) on all days of collection. Turk [28] suggests that during coccidial infections, loss of plasma proteins decreases the effect of absorption of nutrients by the bird. Similar results were obtained by Chapman et al. [26], when working with 10^5^ oocysts of* E. maxima*, who observed that the birds showed a slight hypoproteinemia in the acute phase of the disease (4° dpi), not returning to its normal level until the 10th dpi. Being the liver, the responsible organ for producing albumin, normal concentrations of this protein found in the present study are expected since the parasite does not cause damage to that organ. Significant differences (*P* < 0.05) were observed in experimental animals in triglycerides only in dpi 25° and 25° in total cholesterol and 35 dpi. Coccidiosis is one of the diseases that causes more reductions in plasma lipids due to alterations of intestinal mucosal epithelium [9]. A significant reduction in the absorption of triglycerides was accompanied by a reduction in total cholesterol, which showed the lowest average in infected group, although insignificant. This shows that the dose of* E. maxima* caused reduction of lipid absorption. Similar results were found by Allen and Fetterer [9], who observed a decrease in the absorption of lipids in birds infected with* E. maxima*.

Freitas et al. (2008), investigating the changes in metabolism in chickens experimentally infected with* acervulina*, enteric parasite, noted that all classes of lipids in serum and lipoproteins showed were reduced. Pathologic evaluation showed congested mucosa and the presence of large amounts of mucus in the 6th dpi. The transverse incision of the jejunum enabled the observation of hemorrhagic mucosa featuring the main area affected by the parasite. Microhemorrhages were observed only in the serous 1 dpi, being found mainly in the duodenum and proximal jejunum third region. In the present study, the control group showed no clinical alterations, nor lesions suggestive of infection.

Using a dose of 2 × 10^4^ sporulated oocysts of* E. maxima*, Zulpo [13] also observed scores of moderate injury. Jenkins et al. [29] also observed moderate average scores of 1.9, using a lower dose of 10^3^ oocysts of* E. maxima*. However, different results were obtained by Galha et al. [30], who found scores of moderate injury, but in naturally infected birds. Galha et al. [30], studying the clinical coccidiosis in broiler chickens naturally infected, noted that the main clinical signs were diarrhea and depression, associated with the presence of oocysts of* E. maxima* feces. Santos [5], studying the occurrence of* E. maxima* in naturally infected birds, observed lesions characterized by petechiae on the jejunum serosa, mucosal hyperemia, and orange colored mucous. Jenkins et al. [29] using the 10^4^ dose of oocysts of* E. maxima* observed that birds had lesions and numerous hemorrhages in the serosa, intestinal wall thickening, and distention.

According to Yun et al. [31], the magnitude of clinical signs resulting from infection with* Eimeria* spp. is shaped by factors such as age, sex, lineage, nutrition, and genetic factors of the host. In industrial production of broilers, the mortality is not the biggest problem caused by coccidiosis, but the reduction of the levels of performance caused by diarrhea and sometimes anemia [32]. These results show that it is possible that isolation of* E. maxima* vary according to their ability to induce intestinal lesions [12, 33].

In the infected group, the histopathological examination revealed increased inflammation in the intestinal villi characterized by diffuse lymphocytic infiltrate in the lateral and apical regions of the villi associated with atrophy of the villi and presence of various parasitic forms of* E. maxima*. Histopathological lesions are similar to the lesions found by Zulpo [13], where birds induced 2 × 10^4^ sporulated oocysts of* E. maxima* and had mild villous atrophy, epithelial cell proliferation, and mild hemorrhage in various stages of parasite development [22, 34].

The pathogenic effects of parasitism include growth retardation and reduced feed efficiency [12, 34]. All injuries resulting from parasitic reproduction directly interferes with the absorption of nutrients, triggering a series of disturbances in the metabolism of carbohydrates, lipids, proteins, and macro- and microminerals [35].

## 4. Conclusions

Based on the results obtained, it can be concluded that the dose of 5 × 10^4^ *E. maxima* inoculated in the experimental group was enough to cause damage to the animal organism with no significant difference in relation to growth performance, although the production of chicken meat has been higher in the control group. Infection caused by* E. maxima* has had significance on biochemical examinations related to proteins, total cholesterol, and triglyceride levels in birds from the control group in recent dpi, not being exceeded during the entire period of the experiment of the chickens in the infected groups. During the experimental period no changes in hematocrit levels in both experimental groups were observed. Pathologic evaluation showed congested mucosa and the presence of large amounts of mucus in the 6th dpi.

It is suggested to conduct further studies to test the safety and effectiveness of the results that were not statistically significant and the implementation of studies involving drugs, bioactive, or probiotics as methods of treating infected animals.

## Figures and Tables

**Table 1:** Zootechnical parameters for Coob male broilers-control group and the one infected with sporulated oocysts of *E. maxima*.

	Experimental groups				
Experiencing days	Control		Infected		*P* ^*^
	Average	SD	Average	SD	
10° dpi					
Live weight	386,67	57,26	365,13	45,49	0,264
Carcase weight	274,47	42,34	265,60	48,66	0,599
Heart weight	2,40	0,33	2,50	0,34	0,432
Liver weight	16,24	3,22	17,14	2,96	0,430
Gizzard weight	13,16	1,69	12,62	0,96	0,287
15° dpi					
Live weight	493,33	56,32	451,42	71,34	0,100
Carcase weight	365,00	46,92	328,83	56,57	0,081
Heart weight	3,00	0,32	2,73	0,53	0,123
Liver weight	13,42	1,57	16,97	3,02	***0,001***
Gizzard weight	13,68	1,60	13,09	1,89	0,389
25° dpi					
Live weight	633,80	62,79	668,47	71,34	0,169
Carcase weight	473,13	47,86	485,87	56,57	0,511
Heart weight	3,47	0,48	3,81	0,48	0,062
Liver weight	25,04	4,00	23,85	3,91	0,418
Gizzard weight	17,82	1,96	18,39	2,70	0,517

∗Student's *t*-test; SD = standard-deviation.

The value of “*P*” em bold italic indicates a statistical difference of the level of 5%.

**Table 2:** Haematological and clinical biochemistry variables for Coob male broilers-control group and the one infected with sporulated oocysts of *E. maxima*.

	Experimental groups				
Experiencing days	Control		Infected		*P* ^*^
	Average	SD	Average	SD	
5° dpi					
Glucose	209,17	38,70	173,52	33,36	***0,012***
Triglyceride	122,66	57,47	121,25	46,81	0,942
Total cholesterol	104,77	40,39	130,31	102,78	0,378
Total protein	3,37	0,52	2,61	0,39	***<0,001***
Haematocrit	—	—	—	—	—
10° dpi					
Glucose	231,24	25,33	206,59	26,01	***0,014***
Triglyceride	192,96	87,02	181,12	67,23	0,680
Total cholesterol	126,71	32,28	133,49	46,04	0,644
Total protein	3,58	0,70	2,50	0,19	***<0,001***
Haematocrit	29,35	1,47	27,88	2,92	0,163
15° dpi					
Glucose	233,73	39,10	197,68	22,48	***0,004***
Triglyceride	135,67	62,13	132,21	45,99	0,864
Total cholesterol	130,36	27,39	119,57	29,29	0,306
Total protein	3,44	0,43	2,50	0,36	***<0,001***
Haematocrit	28,96	2,71	29,00	2,49	0,976
25° dpi					
Glucose	266,59	72,09	139,52	32,92	***<0,001***
Triglyceride	116,50	16,06	100,56	17,60	***0,022***
Total cholesterol	137,92	17,04	119,86	14,06	***0,006***
Total protein	3,46	0,57	2,57	0,24	***<0,001***
Haematocrit	28,25	2,17	28,63	3,10	0,721
35° dpi					
Glucose	252,92	35,14	121,85	35,45	***<0,001***
Triglyceride	111,14	24,07	101,10	17,67	0,220
Total cholesterol	148,47	16,21	135,24	13,37	***0,026***
Total protein	2,99	0,35	2,52	0,29	***0,001***
Haematocrit	29,30	3,47	31,46	3,44	0,111

∗Student's *t*-test; SD = standard-deviation.

The value of “*P*” em bold italic indicates a statistical difference of the level of 5%.
